# Infectious Diseases in Children and Body Mass Index in Young Adults

**DOI:** 10.3201/eid1809.111821

**Published:** 2012-09

**Authors:** Gina Suh, Catherine Ley, Julie Parsonnet

**Affiliations:** Stanford University School of Medicine, Stanford, California, USA

**Keywords:** infectious diseases, obesity, body mass index, upper respiratory tract infection, childhood infections, unsanitary conditions

## Abstract

In a cohort of 1,863 Filipinos, diarrhea, fever, and unsanitary conditions in infancy were associated with a decreased body mass index in adulthood; upper respiratory tract infection was associated with an increased body mass index. These finding support the hypothesis that infections early in life play a role in body habitus in adulthood.

Obesity rates are increasing globally, particularly in countries transitioning from a state in which mortality rates are driven primarily by infectious disease to a state in which mortality rates are driven by chronic diseases. In the Philippines, a shift in obesity rates occurred between 1985 and 2005, with weight increasing in 40-year-old women by 9.6 kg (*1*). By 2011, 24.6% of Filipino men and 28.4% of women were overweight (*2*).

Rapid changes in population health historically have been caused by the elimination or introduction of infectious pathogens. In the last 3 decades, infectious diseases among humans have been disappearing from society because of vaccines and improved vaccination practices (*3*), widespread antimicrobial drug use, expanded medical care access, and improved sanitation (*4*). Infections cause alterations in inflammatory cytokines and adipocytokines and raise metabolic rates, and they may influence the colonizing microbiota, which vary with the colonized person’s weight (*5*). Childhood infections may thus affect a person’s long-term weight. We sought to determine whether infectious disease prevalence in infancy predicted body mass index (BMI) in adulthood.

## The Study

We analyzed data from the Cebu Longitudinal Health and Nutrition Survey, an ongoing study of a cohort of Filipino women who gave birth between May 1, 1983, and April 30, 1984, in Cebu, the Philippines (www.cpc.unc.edu/projects/cebu) (*6*). Our study included data from children who were born and reached young adulthood during 1983–2005. Anthropometric, dietary, and infectious disease symptom data were collected bimonthly during the first 24 months of life for children born during that time. Weight and height measurements were again obtained when participants were 22 years of age.

Childhood infectious disease prevalence was derived from symptoms reported at the 12 bimonthly visits. The prevalence for each child was calculated as follows: the proportion of visits when the child had a current fever, the proportion of visits when the child had a cough or nasal congestion within the prior week (upper respiratory infection [URI]), the proportion of visits when the child had diarrhea within the prior week, and the total number of days with diarrhea within the prior week. The child’s highest educational level attained as an adult was used as a marker for adult socioeconomic status (SES). The mother’s highest number of years of schooling or vocational training was used as a partial surrogate for childhood SES; other childhood SES markers included household crowding, toilet type, and presence of animal or human excrement around the home. For inclusion in models, we additionally selected a priori environmental factors likely to be correlated with infectious illness: water source and the proportion of visits in which the child was observed crawling in unsanitary conditions (i.e., in a yard containing excrement).

Outcomes included BMI and whether the person was considered “overweight” or “obese” (for Asian populations, BMI >23 kg/m^2^ and >27 kg/m^2^) (*7*). Median values for continuous variables and proportions for discrete variables were calculated overall and by overweight category; differences between groups were assessed by using a median or χ^2^ test. Pearson coefficient was used to determine coefficient.

Linear regression was used to determine whether childhood infectious disease prevalence independently predicted increased BMI in adulthood. Logistic regression was used to examine the association between infectious disease in a child and an outcome of the person being overweight as an adult. We also explored the effect of infectious disease on BMI at age 24 months. All SES and potential exposure variables were also included in all models, as were birth weight and sex (*8*,*9*). Variables other than those for infectious disease prevalence were retained only when p<0.05.

A total of 1,893 participants had a measurable BMI reported as adults; of these, 1,863, who had at least 1 visit recorded during their first 24 months of life, were included in analyses (Table 1). Few of the adults were obese (3.1%), and 21.2% had >12 years of schooling (data not shown). Conditions during infancy reflected high levels of poverty.

**Table 1:** Distribution of demographic characteristics, infectious disease incidence, and exposure variables during childhood and their association with being overweight in young adulthood, the Philippines, 1983–2005*†

Variable	Overall	Not overweight, <23 kg/m^2^	Overweight, >23 kg/m^2^	p value‡
No. (%) participants	1,863	1,520 (81.6)	343 (18.4)	
Adult BMI, kg/m^2^				
Mean	20.73			
Median	20.16			
25th–75th percentile	18.7–22.2			
Range	13.9–41.2			
No. assessment visits performed during child’s first 24 mo of life				
Mean	11.52	11.5	11.5	0.34
Median	12	12	12	
25th–75th percentile	12–12	12–12	12–12	
Range, no. (%)	12–1	12–1	12–1	
10–12	1,748 (93.8)	1,427 (93.9)	321 (93.6)	0.84
1–9	115 (6.2)	93 (6.1)	22 (6.4)	
Proportion of visits with current fever§				
Mean	0.07	0.07	0.07	0.57
Median	0.08	0.08	0.08	
25th–75th percentile	0–0.08	0–0.08	0–0.83	
Range percentile, no. (%)	0–0.70	0–0.70	0–0.33	
0–19	1,721 (92.4)	1,405 (92.4)	316 (92.1)	0.03¶
20–39	137 (7.4)	110 (7.2)	27 (7.9)	
40–59	4 (0.2)	4 (0.3)	0	
60–79	1 (0.1)	1 (0.1)	0	
80–100	0	0	0	
Proportion of visits with reported URI§				
Mean	0.72	0.72	0.74	0.05
Median	0.75	0.75	0.75	
25th–75th percentile	0.58–0.88	0.58–0.88		
Range percentile, no. (%)	0–1	0–1		
0–19	24 (1.3)	21 (1.4)	3 (0.9)	0.26
20–39	78 (4.2)	68 (4.5)	10 (2.9)	
40–59	426 (22.9)	358 (23.6)	68 (19.8)	
60–79	553 (29.7)	447 (29.4)	106 (30.9)	
80–100	782 (42.0)	626 (41.2)	156 (45.5)	
Proportion of visits with reported diarrhea§				
Mean	0.19	0.19	0.19	0.84
Median	0.17	0.17	0.17	
25th–75th percentile	0–0.25	0.08–0.25	0.83–0.25	
Range percentile, no. (%)	0–1	0–1	0–0.67	
0–19	1,135 (61.0)	928 (61.1)	207 (60.3)	<0.001¶
20–39	507 (27.2)	416 (27.4)	91 (26.5)	
40–59	200 (10.7)	160 (10.5)	40 (11.7)	
60–79	17 (0.9)	12 (0.8)	5 (1.5)	
80–100	4 (0.2)	4 (0.3)	0	
Duration of diarrhea, d§				
Mean	3.42	3.39	3.58	0.69
Median	2.00	2.0	2.0	
25th–75th percentile	0–5	0–5	0–6	
Range	0–24	0–24	0–21	
Proportion of visits when child was crawling in unsanitary conditions				
Mean	0.33	0.34	0.31	0.13
Median	0.27	0.33	0.25	
25–75%	0.08–0.56	0.08–0.58	0.08–0.50	
Range	0–1	0–1	0–0.92	
Male sex, no. (%)	980 (52.6)	789 (51.9)	191 (55.7)	0.14
Highest grade attained, y				
Mean	10.41	10.34	10.68	0.21
Median	11.00	11	11	
25–75%	9–12	9–12	9–12	
Range	0–16	0–16	16–1	
Birth weight, kg				
Mean	3.04	3.04	3.09	0.18
Median	3.00	3.00	3.06	
25th–75th percentile	2.72–3.31	2.72–3.32	2.78–3.36	
Range	0.91–5.21	0.91–5.22	1.96–4.80	
BMI at age 24 mo				
Mean	15.58	15.51	15.89	<0.001
Median	15.54	15.48	15.94	
25th–75th percentile	14.78–16.36	14.73–16.28	15.09–16.64	
Range	11.99–20.62	11.99–19.92	12.28–20.62	
Persons per room				
Mean	2.53	2.52	2.45	0.15
Median	2.00	2.0	2.0	
25th–75th percentile	2.49–3.31	1.50–3.00	1.40–3.00	
Range	0.90–5.21	0.33–13.00	0.33–10.00	
Mother’s schooling or training, y§				
Mean	7.49	7.26	8.50	<0.001
Median	6.00	6.00	8.00	
25–75%	5.00–10.00	5–10	6–11	
Range	0–19	0–18	0–19	
Feces observed around the home, no. (%)	902 (48.4)	735 (48.4)	167 (48.7)	0.96
Covered source of water, no. (%)	1,549 (83.1)	1,242 (81.7)	307 (89.5)	<0.001
Flush toilet, no. (%)	97 (5.2)	73 (4.8)	24 (7.0)	0.11

*BMI, body mass index; URI, upper respiratory infection. Blank spaces indicate not applicable. †The young adults were ≈22 y. ‡Test of proportions or of medians, comparing groups that were not overweight and those that were overweight. §The correlation between the number and the proportion of visits for each symptom was high (coefficient range: 0.86 [URI] to 0.99 [fever, ear discharge]). The correlation between proportion of visits when child had reported fever, URI, and/or diarrhea was moderate (coefficient range: 0.17–0.25). The correlation between the proportion of visits when diarrhea was reported and the number of reported days with diarrhea was high (r = 0.73). Maternal educational level was not well correlated with child’s reported fever, URI, or diarrhea (coefficient range: –0.07 to –0.11). ¶Fisher exact test.

Subjects participated in ≈11.5 visits during the first 24 months of life (Table 1). Cough and nasal discharge or congestion were common (reported at a mean of 6.85 and 7.09 visits, respectively); ear discharge and sore throat were rare (mean 0.36 and 2.37 visits, respectively). Reported fever during the visit was unusual (mean 0.82 of 12 visits, respectively); fever reported before the visit or ongoing diarrhea was slightly more common (mean 2.19 of 12 visits, respectively). The mean reported number of days of diarrhea in the 12 sampling weeks was equivalent to 30 days over a 2-year period (Table 1).

Using multivariate regression modeling, we examined BMI when the participants were 24 months of age and again when participants were 22 years of age (Table 2). We identified reported diarrhea, crawling in unsanitary conditions, and URI in infancy as predictors of BMI at age 22 years. The individual effects of these factors, however, were opposing: diarrhea and crawling in unsanitary conditions were associated with decreased adult BMI (p = 0.06 and p = 0.02, respectively), and URI was associated with increased adult BMI (p = 0.01). Point estimates for the proportion of visits with fever, although not statistically significant, showed a trend toward decreased BMI. Significant predictors of BMI at 24 months included only proportion of visits with fever, which was associated with a decreased BMI. No interactions between infectious disease variables and other predictors were identified. Logistic regression modeling of overweight adults (BMI >23 kg/m^2^) identified similar findings (data not shown). Again, reported URI in children was associated with higher prevalence of overweight adults, whereas crawling in unsanitary conditions in infancy was linked to decreased prevalence of overweight adults. The point estimates for both the number of visits with current fever and with reported diarrhea, although not statistically significant, showed that these factors were protective against being overweight in adulthood (Table 2).

**Table 2:** Independent predictors of BMI in a cohort of persons during childhood and during adulthood, the Philippines, 1983–2005 *†

Predictor	BMI, age ≈24 mo			BMI, age ≈22 y	
	Parameter estimate	p value		Parameter estimate	p value
Visits with reported URI‡	–0.25	0.14		1.13§	0.01
Visits with current fever	–1.41	<0.001		–1.61	0.11
Visits with reported diarrhea	0.23	0.27		–1.05	0.06
Visits during which child was observed crawling in unsanitary conditions	–0.05	0.65		–0.72	0.02

*BMI, body mass index; URI, upper respiratory infection. †Models were adjusted for maternal schooling/training (<7 or >7 y), sex and birth weight. R^2^: BMI age 24 mo, 0.08; BMI age 22 y, 0.03. ‡A model that used number of visits instead of proportion of visits identified very similar findings, as did a model that limited analysis to those with >10 visits only. §The parameter estimate corresponds to the change in BMI when participants with no visits (0%) with infection are compared to those with all visits (100%) with infection. Alternatively, a 10% increase in reported visits with infection corresponds to an increase of 10% of the parameter in BMI in adulthood. For example, a 10% increase in visits with reported URI corresponds to a 0.113 kg/m^2^ increase in adult BMI.

## Conclusions

In an economically disadvantaged Filipino population, we observed that frequency of diarrhea in infancy was associated with a lower BMI in adulthood. Time spent crawling in unsanitary conditions—a surrogate for SES and exposure to pathogenic fecal bacteria carried by humans—was also linked to BMI in adulthood. Cumulative infectious diarrheal diseases in children are known to affect their growth and development (*10*). Although this effect on growth may be because of decreased nutrient absorption, a more enduring consequence may be the modification of the gut microbiome (*11*). For example*, Helicobacter pylori*, a common component of the microbiome in populations with decreased household sanitation, has been associated with decreased adult weight (*12*). Studies of the colonic microbiota are under way to determine whether their establishment in childhood predicts adult adioposity.

In contrast to the frequency of diarrheal diseases, URI frequency was associated with increased BMI or a score that indicates that the person is overweight. This association ran counter to our original hypothesis that infectious illness in early infancy is associated with decreased BMI in adults. Researchers have postulated that exposure to specific respiratory pathogens (e.g., adenovirus 36) may increase weight (*13*). No consensus exists, however, regarding the importance of these individual pathogens, which may simply identify markers for other exposures. Another possible explanation for the opposite effects of respiratory and gastrointestinal infections may be that respiratory infections lead to antimicrobial drug use, (which has, itself, been linked to higher weight in animals and in humans [*14*]), whereas the gastrointestinal infections do not.

The Cebu study was largely a study of nutrition; infection was of only minor interest as a confounder. As such, infection relied on maternal recall without detailed temperature measurements or confirmatory diagnoses. Yet, despite the fact that the database was not designed with infectious disease exposures as a primary measure, we were able to identify a role of infection in BMI after adjusting for multiple markers of SES. Although childhood infections may be a surrogate for unmeasured characteristics that correlate with adult BMI levels, we were unable to identify these confounders despite the many covariates we assessed.

In summary, our study demonstrated an association between frequency of symptomatic infectious diseases during infancy and BMI in adults. Prospective studies in well-characterized subjects are needed to confirm these findings, delineate mechanisms, and assess the magnitude of the effects of early childhood infectious diseases on weight.
